# Correction: Chen et al. Secondary Metabolites with Anti-Inflammatory from the Roots of *Cimicifuga taiwanensis*. *Molecules* 2022, *27*, 1657

**DOI:** 10.3390/molecules27186035

**Published:** 2022-09-16

**Authors:** Jih-Jung Chen, Ming-Jen Cheng, Tzong-Huei Lee, Yueh-Hsiung Kuo, Chao-Tsen Lu

**Affiliations:** 1 Department of Pharmacy, School of Pharmaceutical Sciences, National Yang Ming Chiao Tung University (NYCU), Taipei 112, Taiwan; 2 Department of Medical Research, China Medical University Hospital, China Medical University, Taichung 404, Taiwan; 3 Bioresource Collection and Research Center (BCRC), Food Industry Research and Development Institute (FIRDI), Hsinchu 300, Taiwan; 4 Institute of Fisheries Science, National Taiwan University, Taipei 10617, Taiwan; 5 Department of Chemistry, National Taiwan University, Taipei 106, Taiwan; 6 Department of Biotechnology, Asia University, Taichung 413, Taiwan; 7 Department of Chinese Pharmaceutical Sciences and Chinese Medicine Resources, College of Pharmacy, China Medical University, Taichung 404, Taiwan; 8 Chinese Medicine Research Center, China Medical University, Taichung 404, Taiwan

The authors wish to make the following changes to their paper [1]. 

## Author List

**Original:**

Jih-Jung Chen ^1,2^, Ming-Jen Cheng ^3,^*, Tzong-Huei Lee ^4^, Yueh-Hsiung Kuo ^5,6,7,^* and Chao-Tsen Lu ^5^

^1^ Department of Pharmacy, School of Pharmaceutical Sciences, National Yang Ming Chiao Tung University (NYCU), Taipei 112, Taiwan

^2^ Department of Medical Research, China Medical University Hospital, China Medical University, Taichung 404, Taiwan

^3^ Bioresource Collection and Research Center (BCRC), Food Industry Research and Development Institute (FIRDI), Hsinchu 300, Taiwan

^4^ Institute of Fisheries Science, National Taiwan University, Taipei 10617, Taiwan

^5^ Department of Chemistry, National Taiwan University, Taipei 106, Taiwan

^6^ Department of Biotechnology, Asia University, Taichung 413, Taiwan

^7^ Department of Chinese Pharmaceutical Sciences and Chinese Medicine Resources, College of Pharmacy, China Medical University, Taichung 404, Taiwan

* Correspondence: chengfirdi@gmail.com (M.-J.C.); yhkuo800@gmail.com (Y.-H.K.)

**To be replaced with:**

Jih-Jung Chen ^1,2,†^, Ming-Jen Cheng ^3^, Tzong-Huei Lee ^4^, Yueh-Hsiung Kuo ^5,6,7,8,^* and Chao-Tsen Lu ^5,†^

^1^ Department of Pharmacy, School of Pharmaceutical Sciences, National Yang Ming Chiao Tung University (NYCU), Taipei 112, Taiwan

^2^ Department of Medical Research, China Medical University Hospital, China Medical University, Taichung 404, Taiwan

^3^ Bioresource Collection and Research Center (BCRC), Food Industry Research and Development Institute (FIRDI), Hsinchu 300, Taiwan

^4^ Institute of Fisheries Science, National Taiwan University, Taipei 10617, Taiwan

^5^ Department of Chemistry, National Taiwan University, Taipei 106, Taiwan

^6^ Department of Biotechnology, Asia University, Taichung 413, Taiwan

^7^ Department of Chinese Pharmaceutical Sciences and Chinese Medicine Resources, College of Pharmacy, China Medical University, Taichung 404, Taiwan

^8^ Chinese Medicine Research Center, China Medical University, Taichung 404, Taiwan

* Correspondence: kuoyh@mail.cmu.edu.tw

† These authors contributed equally to this work.
